# Supplementary material for: A note on the Wilcoxon-Mann-Whitney test and tied observations
Source: PLoS One. 2024 Aug 21;19(8):e0309074. doi: 10.1371/journal.pone.0309074 (PMC11338448; doi:10.1371/journal.pone.0309074)
Supplement: S1 File — (PDF) [file pone.0309074.s001.pdf]

```
# Simulation of actual size and power of the Wilxoxon rank-sum test

# after rounding and after omission of ties

library(coin)
library(VGAM)


Simul.1 <- function(K, shift) {

power1 <- 0; power2 <- 0; power3 <- 0; power4 <- 0
power1a <-0; power2a <- 0; power1b <- 0; power2b <- 0
power3a <- 0; power4a <- 0; number.0 <- 0; number.new <- 0

for (j in 1:K) {

# In the following lines, we simulate data of the different distributions,
# under the null hypothesis for simulation of actual size, or under the
# alternative for simulation of power, and for different sample sizes.
# In each case the code is to be used for one simulation scenario, and
# the code for all other scenarios is commented out.

# random numbers for simulation of actual size
#x1 <- rnorm(20)
#x1 <- rexp(20)
#x1 <- rcauchy(20)
#x1 <- rlaplace(20)

#x1 <- rnorm(21)
#x1 <- rexp(21)
#x1 <- rcauchy(21)
#x1 <- rlaplace(21)

# random numbers for simulation of power
y1 <- rnorm(10)
y2 <- rnorm(10) + shift
#y1 <- rexp(10)
#y2 <- rexp(10) + shift
#y1 <- rcauchy(10)
#y2 <- rcauchy(10) + shift
#y1 <- rlaplace(10)
#y2 <- rlaplace(10) + shift

#y1 <- rnorm(14)
#y2 <- rnorm(7) + shift
#y1 <- rexp(14)
#y2 <- rexp(7) + shift
#y1 <- rcauchy(14)
#y2 <- rcauchy(7) + shift
#y1 <- rlaplace(14)
#y2 <- rlaplace(7) + shift

x1 <- c(y1,y2) # this line is needed for power simulation only

# In the following two lines, we round data to one or two decimal places.
# One line is to be used, the other line is commented out.

x2 <- round(x1,1) # rounding to one decimal place
#x2 <- round(x1,2) # rounding to two decimal places

# For creation of data.a1 the code is different depending on sample size.
# One line is to be used, the other line is commented out.
```

```
# creation of a data frame for two groups with 10 observations
data.a1 <- data.frame(x1, x2, dose=factor(rep(1:0,rep(10,2))))

# creation of a data frame for two groups with 14 and 7 observations
#data.a1 <- data.frame(x1,x2,dose=factor(c(rep(1,7), rep(1:0,rep(7,2)))))

# Omission of ties
doppelt <- unique(x2[duplicated(x2) == TRUE])
data.unique <- subset(data.a1, !(x2 %in% doppelt))


length0 <- length( subset(data.unique, dose==0)$x2 )
length1 <- length( subset(data.unique, dose==1)$x2 )


number.new <- number.new + length(data.unique$x2)



# exact Wilcoxon test with original data
p.value1 <- pvalue(wilcox_test(x1~dose, data=data.a1,
distribution="exact"))

# asymptotic Wilcoxon test with original data
p.value2 <- pvalue(wilcox_test(x1~dose, data=data.a1,
distribution="asymptotic"))

# exact Wilcoxon test with rounded data
p.value1a <- pvalue(wilcox_test(x2~dose, data=data.a1,
distribution="exact"))   #exact WMW

# asymptotic Wilcoxon test with rounded data
p.value2a <- pvalue(wilcox_test(x2~dose, data=data.a1,
distribution="asymptotic"))   #asy. WMW

# Counting of data sets with at least one empty group
if (min(length0, length1)==0) {number.0 <- number.0 +1}

if (min(length0, length1)==0) {p.value3=NA}

else

# exact Wilcoxon test after omission of ties, alpha = 0.05
{if (pvalue(wilcox_test(x2~dose, data=data.unique, distribution="exact"))
<= 0.05)

{power3 <- power3 + 1}}   #exact WMW

if (min(length0, length1)==0) {p.value4=NA}

else

# asymptotic Wilcoxon test after omission of ties, alpha = 0.05
{if (pvalue(wilcox_test(x2~dose, data=data.unique,
distribution="asymptotic")) <= 0.05)

{power4 <- power4 + 1}}    #asy. WMW

if (min(length0, length1)==0) {p.value3a=NA}

else

# exact Wilcoxon test after omission of ties, alpha = 0.01
{if (pvalue(wilcox_test(x2~dose, data=data.unique, distribution="exact"))
<= 0.01)

{power3a <- power3a + 1}}   #exact WMW

if (min(length0, length1)==0) {p.value4a=NA}
```

```
else

# asymptotic Wilcoxon test after omission of ties, alpha = 0.01
{if (pvalue(wilcox_test(x2~dose, data=data.unique,
distribution="asymptotic")) <= 0.01)

{power4a <- power4a + 1}}    #asy. WMW


if (p.value1 <= 0.05) {power1 <- power1 + 1}

if (p.value2 <= 0.05) {power2 <- power2 + 1}

if (p.value1a <= 0.05) {power1a <- power1a + 1}

if (p.value2a <= 0.05) {power2a <- power2a + 1}

if (p.value1a <= 0.01) {power1b <- power1b + 1}

if (p.value2a <= 0.01) {power2b <- power2b + 1}

}

number.av <- number.new/K   # average sample size after omission of ties

exact5.orig <- power1/K

asy5.orig <- power2/K

exact5.ties <- power1a/K

asy5.ties <- power2a/K

exact1.ties <- power1b/K

asy1.ties <- power2b/K

exact5.omit <- power3/K

asy5.omit <- power4/K

exact1.omit <- power3a/K

asy1.omit <- power4a/K

power <- c(number.av,number.0,exact5.orig, exact5.ties, exact5.omit,
asy5.orig, asy5.ties, asy5.omit, exact1.ties, exact1.omit, asy1.ties,
asy1.omit)

power

}

set.seed(13579)

Simul.1(10000,1.5)
```
