# Supplementary material for: A note on the Wilcoxon-Mann-Whitney test and tied observations
Source: PLoS One. 2024 Aug 21;19(8):e0309074. doi: 10.1371/journal.pone.0309074 (PMC11338448; doi:10.1371/journal.pone.0309074)
Supplement: Attachment — Submitted filename: Response to reviewers for a note on the WMW test.pdf [file pone.0309074.s002.pdf]

A note on the Wilcoxon-Mann-Whitney test and tied observations

Response to Reviewers

Reviewer #1: This short note concerns the use of the Wilcoxon-Mann-Whitney (WMW) test in the presence of ties. Previously, McGee (2018) recommended removing the ties from analysis on the grounds that they interfere with the normal approximation. In this paper, however, the authors use a simulation to show that removal of ties generally leads to undesirable properties of the test. It is concisely and well written, and I am in favor of publishing it.

As a side note, the idea of keeping all data in the analysis rather than focusing on a subset (in this case untied observations) is captured in the famous intent-to-treat (ITT) principle. Recently, Mao (2022) studied the properties of the WMW test under the ITT principle in the presence of noncompliance. Although this is a slightly different scenario, the authors may consider connecting to this literature to strengthen their case and bring it up to date.

Mao, L. (2022). On the relative efficiency of intent-to-treat Wilcoxon–Mann–Whitney test in the presence of non-compliance. Biometrika, 109, 873-880.

Our reply: Thank you for providing this reference. In the revised version of our manuscript (see page 4) we added the following statement: "In this context please note that Mao (2022) recently investigated the Wilcoxon-Mann-Whitney test in the presence of noncompliance. The noncompliers, included in an intention-to-treat analysis, are not an evenly distributed subset, and Mao (2022) showed that the properties of the Wilcoxon-Mann-Whitney test depend on whether noncompliers are more likely in high-density regions or in the tails of the outcome distribution."

We think that a longer discussion of Mao's findings would not further support our reasoning because Mao (2022) did not compare the intention-to-treat WMW test with a test after omitting noncompliers (i.e. a per-protocol WMW test). Instead, Mao (2022) did not omit observations and compared the intention-to-treat WMW test with the intention-to-treat t test. However, the comparison of the WMW test versus the t test is not our focus.

Minor:

"In is true that" -> "It is true that"

Our reply: Corrected.

In addition, Reviewer #1 replied “no” at question 3 (Have the authors made all data underlying the findings in their manuscript fully available?)

Our reply: We provide the simulation program so that all our data can be reproduced. Thus, in our opinion, and in the opinion of Reviewer #2, we made all data underlying our findings fully available. Nevertheless, we added further comments within our R code for simulation to enable better understanding.

Reviewer #2: Overall this is a clear and concise paper that makes a very clear point about not omitting ties when using the Wilcoxon-Mann-Whitney test. The limitations and flaws of the conclusions from McGee 2018 are clear. The only suggestion I would have pertains to something that is not clear to me about McGee's methodology, even after having read their paper. It is not clear how McGee achieved uniformly distributed ties, even after having read the paper. Perhaps this is more clear from the code, which I started to examine, but I assume the authors of this paper have had a closer look. The authors make a point of questioning whether McGee's simulation study considered realistic scenarios. I think maybe a little more needs to be said about those (un)realistic scenarios.

Our reply: In the revised version of our manuscript (see page 4) we added the following statement: “In contrast, in McGee’s simulation a given number of values is randomly selected, regarded as ties and omitted (see e.g. the supplementary file SmallSamplePowerOmit.txt, available at https://github.com/MonnieMcGee/ TiesInRankBasedTests/blob/master/R-Code/SmallSamplePowerOmit.txt).”

To be more precise, values for n1, n2, t, d, and nsims are given for the different simulations. Let’s choose n1 = n2 = 20 observations per group with 25% ties, i.e. t = 3 tied observations per group, d = 0 (for simulation of the actual type I error rate under the null hypothesis) and

just one simulated data set (nsims = 1, then we do not need a loop). The R code according to McGee's file SmallSamplePowerOmit.txt is:

```
totalN <- n1 + n2 - 2*t

samp1 <- rnorm(n1, mean=d)

samp2 <- rnorm(n2)

m <- min(n1,n2)

        #Up to here samp1 and samp2 are the two simulated samples.

idx1 <- sample(1:m,t)

idx2 <- sample(1:m,t)

        #Here, the positions of 3 values per group are randomly sampled, e.g. 11, 9 and 6 in
         group 1 (idx1) and 7, 13 and 20 in group 2 (idx2)

samp1 <- samp1[-idx1]

samp2 <- samp2[-idx2]

        #Then, the values at positions 11, 9 and 6 are omitted in samp1, and the values at
         positions 7, 13 and 20 in samp2

         As a consequence, the omitted (tied) observations are evenly distributed across
         samples.
```
