# Supplementary material for: A note on the Wilcoxon-Mann-Whitney test and tied observations
Source: PLoS One. 2024 Aug 21;19(8):e0309074. doi: 10.1371/journal.pone.0309074 (PMC11338448; doi:10.1371/journal.pone.0309074)
Supplement: Attachment — Submitted filename: PONE-D-23-33167R1-Review.pdf [file pone.0309074.s003.pdf]

Review of PONE-D-23-33167R1
May 25, 2024

[1] made a case that omitting tied observations when using the Wilcoxon-Mann-Whitney (WMW) test and the two-sample t-test (TST) is sometimes the best option in terms of Type I and Type II errors. Neuhäuser and Ruxton have recently submitted an article stating that omission of tied observations is not a good idea in terms of Type I and Type II error for the WMW test.

Neuhäuser and Ruxton have 2 main disagreements with [1].

1. The recommendation to omit tied observations has no practical or theoretical justification.
2. Using all the data, including ties, with an exact permutation test provides better results

I will address the second point first, because I agree with Neuhäuser and Ruxton on this point. In the conclusion to her article, [1] states "This observation [about the effect of ties] begs the question of what two-sample test is useful for discrete or ordinal data when ties are present. *One method is to use a permutation test*, (emphasis added) which is a computationally intensive method of calculating the null distribution of all possible differences in means (or medians, or ratios, etc)." For small samples, even a permutation test will have reduced power, but at least keeping all observations in the sample will mitigate this effect somewhat. Even so, it is not clear what happens in the permutation test setting when samples have very different shapes or different variances; however, it is known that Type I and Type II errors for the WMW are sensitive to differing shapes and variances between populations [2].

To address point 1, it has been suggested that the WMW test itself is not practical or realistic [2] (and references within). The WMW, as originally envisioned by Wilcoxon [3], addressed the null and alternative hypotheses $H_0: \mu_1 = \mu_2 \; vs. H_A: \mu_1 = \mu_2 + \delta$, where $\delta \neq 0$ is a shift in the location of one of the distributions. The shift alternative implies that the two distributions must be equal in all respects except for the shift in location. In fact, it has been shown through extensive simulations, that "small differences in variances and moderate degrees of skewness can produce large deviations from the nominal Type I error rate" [2]. I suspect that this is part of the reason that Neuhäuser and Ruxton's results contradict [1]. Their simulations likely produce situations where the shapes and skewness of the two populations being tested violate the shift alternative framework.

Secondly, as Neuhäuser and Ruxton point out, the position of the ties within a sample can make a difference in Type I error for the WMW [4]; therefore, random placement of ties is unrealistic. The result in [4] gives bounds on Pitman efficiencies given the placement of compliers and noncompliers within samples in an intention to treat analysis for both the WMW and the TST. I am glad to know about this result. However, [1] considered that the

position of ties would affect the Type I and Type II errors by randomly distributing ties in terms of their position within a sample. In the context of experimental design, randomization to mitigate the effects of unexamined factors theoretically and practically justified [5]. Randomization is an experimental tool that mitigates the effect of confounding variables that are unknown or unnamed within an experiment.

As quoted in Fisher, 1926 [6]:

> One way of making sure that a valid estimate of error will be obtained is to arrange the plots deliberately at random, so that no distinction can creep in between pairs of plots treated alike and pairs treated differently; in such a case an estimate of error, derived in the usual way from the variation of sets of plots treated alike, may be applied to test the significance of the observed difference between the averages of plots treated differently.

Thirdly, [1] advocates omitting ties only when the percentage of ties in the sample is less than 15%. Figure 5 [1] shows that the WMW has Type I error close to nominal only if the sample sizes are equal, the percentage of ties is 10% or 15%, and only when the the sample sizes for both groups are greater than 10. Therefore, omitting ties should be done when all these stipulations are met.

Lastly, there is a fundamental difference in the way [1] and Neuhäuser and Ruxton perform simulations that indicate inflated Type I error when ties are omitted from the samples. Neuhäuser's and Ruxton's simulations are done in a context where random variables are generated from one of several distributions and then the continuous values are rounded to one decimal place. In their simulations, the WMW test is performed only on the unique values in the data set, which effectively eliminates all tied values. The simulation set up forces the percentage of ties within and between samples to be subject to the observations within each random sample. In essence, the percentage of ties cannot be predicted from the outset, which is the definition of random.

I used Neuhäuser's and Ruxton's available code to perform simulations only for the Normal case where the sample sizes are equal at n = m = 10 and $\mu_1 = \mu_2 = 0$. I used 10000 iterations and the same seed that was provided in the code. My Type I error in the continuous case (all values included) was 0.043 and in the discretized case (ties were present) it was 0.0471, both of which are close to the nominal significance level of 0.05. However, I modified the code somewhat to store the p-values (for both cases), the number of observations kept, and the number of observations in each sample for all 10000 iterations. My modified code is given at the end of this review. A histogram (left) and an empirical distribution function (right) of the percentage of observations deleted, which is equivalent to the percentage of tied observations, is given in Figure 1.

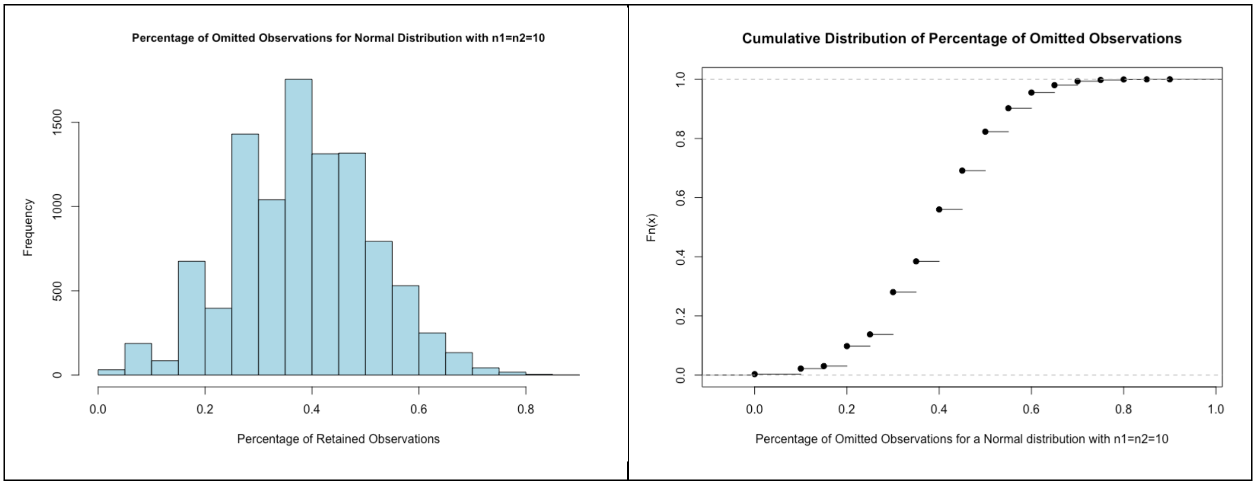

Figure 1: The left panel shows a histogram of the percentage of omitted observations for the case when values from a continuous random normal distribution with mean 0 and variance 1 are rounded to one decimal place, thus forces tied observations. The starting sample size is n = m = 10. The right panel shows an empirical distribution function of the same data.

The center of the histogram is approximately 0.4, which indicates that the average percentage of observations omitted in this simulation is 40%. However, the percentage could be as low a 0% or as high as 90%. The five-number summary for the percentage of tied observations is 0, 30, 40, 50, 90. In other words, at least 25% of the simulations from Neuhäuser and Ruxton have 30% of the observations tied, and at least 75% of them have 50% of the observations tied. This is shown graphically in Figure 1(b) with the empirical distribution function. For the ECDF, the percentage of omitted observations is plotted on the horizontal axis and cumulative proportion for each observation is given on the vertical axis. The simulations have a much greater percentage of tied observations than the simulations in [1], where the percentage of tied observations was kept constant for various scenarios. Neuhäuser and Ruxton need to address this issue in a revision.

Finally, in focusing on the WMW test and the assignment of ties, Neuhäuser and Ruxton miss some of the main ideas of [1]. [1] examined multiple replacements for tied observations, including omission, jittering, mid-ranks, and average scores. Simulations for 15 different pairs of equal sample sizes, ranging from n = m = 5 to n = m = 20, and 14 different sets of unequal sample sizes (starting with n = 6 and m = 3 and ranging to n = 20 and m = 18) were performed. Four types of adjustments for ties were compared using 4 distributions and 4 different percentages of tied observations for both the WMW and the TST. This is a total of $2[(15 \times 4 \times 4 \times 4) + (14 \times 4 \times 4 \times 4)] = 3720$ simulations. These simulations were done for the situation where $\mu_1 = \mu_2 = 0$ (Type I error). Additional simulations were done to determine Type II error. Neuhäuser and Ruxton's results address Type I error and Type II error only for omitting ties, and not for other common methods of correcting for tied observations.

In reviewing this paper, I thought of an additional reason that the placement of ties could affect Type I and Type II error. Perhaps the difference can be thought of as ties "within"

samples vs. ties "between samples". To illustrate the difference, consider the following data set from [1].

> Suppose we have the following data that represent a score on a scale of alertness of 11 students, five who had consumed 8 ounces of coffee prior to the test, and six who drank 8 ounces of water. For the coffee group, we have scores 21 31 29 27 35, and for the water group, we have 21 19 17 21 20 19.

To begin the ranking process, we put all the scores into one vector: **21 31 29 27 35** 21 19 17 21 20 19. The first 5 numbers (in bold red) are from the coffee drinking treatment and the second 5 are from the water drinking treatment. The score of 21 in the coffee treatment and the score of 21 in the water treatment are examples of "between samples" ties, which means that an observation in one sample is tied with an observation in the other sample. The score of 19, appearing twice in the water treatment group, is an example of a within sample tie, because a score of 19 does not appear in the coffee treatment group. Incidentally, the score of 21 is both a between sample tied score and a within sample tied score. [1] focused on between-sample tied scores, as the code randomly selected certain rows with a data frame to be tied, while Neuhäuser and Ruston's simulations contain both within sample and between sample ties.

Of the two results from [1] that Neuhäuser and Ruxton contest, I agree that permutation tests can be used in situations when assumptions for parametric or nonparametric tests are not met. However, permutation tests require either coding ability or the software to perform them, and it is not clear whether many research scientists without statistical training would have access to them. Many research scientists use the tests that are available within Excel or menu-driven statistical software. Neuhäuser's and Ruxton's simulations also have a large percentage of tied observations that are deleted in their simulations. [1] recommended that omitting ties is valid only if the percentage of ties is less than 15% and the sample size is reasonable. The percentage of tied observations was held constant across simulations while varying other factors to examine the effects of each factor separately. Further, random placement of ties has justification in the experimental design literature [6] for mitigating the effect of the placement of ties within the sample.

Minor:

It is easier to compare Type I and Type II errors across multiple samples when the results are plotted in an effective visualization than it is compare numbers across tables. I would like to see Neuhäuser's and Ruxton's tables presented graphically. Not only would a visualization make it easier to compare responses within their study, but also with the results of other similar studies.

*Page 5, line 90 states, "... is estimated as the number of significance tests divided by the number of performed tests", I think the authors meant "significant" tests. To be more precise, the sentence should read, "estimated as the number of **significant** tests for which the p-value is less than the nominal level of significance divided by the number of performed tests", or, even better "estimated*

as the number of tests for which the p-value is less than the nominal level of significance divided by the number of performed tests".

Bibliography

[1] M. McGee, "Case for omitting tied observations in the two-sample t-test and the Wilcoxon-Mann-Whitney Test," *PLoS One,* p. https://doi.org/10.1371/journal.pone.0200837, 2018.

[2] M. W. Fagerland and L. Sandvik, "The Wilcoxon-Mann-Whitney test under scrutiny," *Statistics in Medicine,* vol. 28, pp. 1487-1497, 2009.

[3] F. Wilcoxon, "Individual comparisons by ranking methods," *Biometrics,* vol. 1, pp. 80-83, 1945.

[4] L. Mao, "On the relative efficiency of the intent-to-treat Wilcoxon-Mann-Whitney test in the presence of noncompliance," *Biometrika,* vol. 109, no. 3, pp. 873-880, 2021.

[5] P. Armitage, "Fisher, Bradfor Hill, and Randomization," *International Journal of Epidemiology,* vol. 32, no. 6, pp. 925-928, 2003.

[6] R. A. Fisher, "The arrangement of field experiments," *J Ministry of Agriculture of Great Britain ,* vol. 33, pp. 503-513, 1926.

Modified Code for Figure 1:

```
library(coin)
library(VGAM)

Simul.1 <- function(K){
  p.value1 <- numeric(K)
  p.value1a <- numeric(K)
  sskeep <- numeric(K)
  length0 <- numeric(K)
  length1 <- numeric(K)
  for (j in 1:K){
    x1 <- rnorm(20)
    x2 <- round(x1,1)
  data.a1 <- data.frame(x1, x2, dose=factor(rep(1:0,rep(10,2))))
  doppelt <- unique(x2[duplicated(x2) == TRUE])
  data.unique <- subset(data.a1, !(x2 %in% doppelt))
  length0[j] <- length( subset(data.unique, dose==0)$x2 )
  length1[j] <- length( subset(data.unique, dose==1)$x2 )
  p.value1[j] <- pvalue(wilcox_test(x1~dose, data=data.a1,
                              distribution="exact"))
  p.value1a[j] <- pvalue(wilcox_test(x2~dose, data=data.a1,
                               distribution="exact")) #exact WMW
  sskeep[j] <- length(data.unique$x2)
  # exact Wilcoxon test after omission of ties, alpha = 0.05
}
```

```
return(list(p.value1=p.value1,p.value1a=p.value1a,sskeep=sskeep,length0=lengt
h0,length1=length1))
}

set.seed(13579)
test <- Simul.1(10000)
## Calculations for paper
mean(test$p.value1 < 0.05)
mean(test$p.value1a < 0.05)
hist(1-(test$sskeep/20), main="Percentage of Omitted Observations for Normal
Distribution with n1=n2=10",cex.main=.95, xlab="Percentage of Retained
Observations",col="lightblue")
plot(ecdf(1-(test$sskeep/20)), main="Cumulative Distribution of Percentage of
Omitted Observations",xlab="Percentage of Omitted Observations for a Normal
distribution with n1=n2=10")
summary(1-(test$sskeep/20)) # 5 number summary plus mean of percentage of
omitted values

## Not put in paper
hist(test$length0)
hist(test$length1)
hist(test$length0/test$length1)
summary(test$length0/test$length1)
```
