# Supplementary material for: A note on the Wilcoxon-Mann-Whitney test and tied observations
Source: PLoS One. 2024 Aug 21;19(8):e0309074. doi: 10.1371/journal.pone.0309074 (PMC11338448; doi:10.1371/journal.pone.0309074)
Supplement: Attachment — Submitted filename: Reply 3 to A note on the Wilcoxon.docx [file pone.0309074.s004.docx]

**A note on the Wilcoxon-Mann-Whitney test and tied observations**

**Response to Editor and Reviewer**

Additional Editor Comments:

Please address the useful comments made by the reviewers. Please note that the third reviewer is the author of the original article; while I think she brings up some valid concerns, it seems as if it comes down to some qualifiers/caveats as to when exclusion of ties might be useful. Furthermore, she makes that point that in the original article other methods of dealing with ties was explored (and not simply removal). Improved discussion of these caveats and other contributions of the original work would better couch/place the criticism of the current manuscript within the previous work.

Our reply: Thank you for obtaining this valuable third review. We address all the issues raised in the review in detail below, however for convenience we summarise the key changes made below. Overall, we do not feel that our fundamental recommendations need to change in response to the points raised by the reviewer. However, we have substantially changed the tone of our abstract and introduction so as to be less overly critical of McGee’s original work.

In our key changes in this revision we have added some details of McGee’s simulation, and clarified our conclusions. Specifically, we now write that, according to McGee’s results “… one might advocate omitting ties only when the percentage of ties in the sample is less than 15%. However, such a strategy would often preclude defining the statistical approach ahead of data collection, since the percentage of ties would normally be difficult to predict with any confidence. Further, the exact test with tied observations included has preferable type I error and power values than the test performed after omission of ties in the scenarios presented in Tables 1 and 3 where the average proportion of ties is not larger than 10%. Thus, the exact permutation test is always a good choice. This test is available in various statistical software packages including the open-source statistical software R. In addition, a free and easy-to-use online calculator that provides the exact permutation Wilcoxon-Mann-Whitney test is available at <https://ccb-compute2.cs.uni-saarland.de/wtest/> (Marx et al., 2016).” (see page 6 of the revised version).

Therefore, in our opinion, we do not see any situation when exclusion of ties might be useful.

We also added in the revision (see page 7) “that McGee (2018) also investigated other methods of handling ties. Her detailed study shows some advantages and disadvantages of the various procedures. McGee (2018) concluded that, aside from omission of ties observations, the next best option would be jittering the data, i.e. adding random noise to the observations to break ties.”

Reviewer #3:

I have given a detailed response in an attached PDF. In brief, I believe that the author's results are different from McGee 2018 partially because their percentage of tied observations varies widely between simulations. Also, I agree that permutation tests are a viable option when there are large numbers of ties between or within samples.

Our reply can be found below within the detailed response.

[1] made a case that omitting tied observations when using the Wilcoxon-Mann-Whitney (WMW) test and the two-sample t-test (TST) is sometimes the best option in terms of Type I and Type II errors. Neuhäuser and Ruxton have recently submitted an article stating that omission of tied observations is not a good idea in terms of Type I and Type II error for the WMW test.

Neuhäuser and Ruxton have 2 main disagreements with [1].

1. The recommendation to omit tied observations has no practical or theoretical

justification.

2. Using all the data, including ties, with an exact permutation test provides better

results

I will address the second point first, because I agree with Neuhäuser and Ruxton on this point. In the conclusion to her article, [1] states “This observation [about the effect of ties] begs the question of what two-sample test is useful for discrete or ordinal data when ties are present. One method is to use a permutation test, (emphasis added) which is a computationally intensive method of calculating the null distribution of all possible differences in means (or medians, or ratios, etc).”

Our reply: We are glad that the reviewer is in agreement with us. In the revised version we now say (see pages 6-7) that “the exact permutation test is always a good choice … When applying this permutation test there is, even in the case of discrete or ordinal data, no need, and no reason, to replace the test statistic. Thus, the permutation test should be performed with the Wilcoxon rank sum, not with the difference in means, medians, or ratios. The exact permutation Wilcoxon-Mann-Whitney test can be applied even in the extreme case of binary data: when two groups are compared based on binary data the standard method is Fisher’s exact test; this test can be considered as a special case of the exact version of the Wilcoxon-Mann-Whitney test; both tests result in identical p-values (Brunner et al., 2019, p. 106).”

For small samples, even a permutation test will have reduced power, but at least keeping all observations in the sample will mitigate this effect somewhat.

Our reply: Our simulations also show that an asymptotic test can have a larger power than an exact permutation test for small sample sizes. However, this effect is not consistent and an asymptotic test should be avoided anyway in the case of small samples because the validity of the asymptotic approximation is doubtful for small data sets (for more details see Neuhäuser and Ruxton (2024): The statistical analysis of small data sets. Oxford University Press). We make this point clearly in the manuscript.

Even so, it is not clear what happens in the permutation test setting when samples have very different shapes or different variances; however, it is known that Type I and Type II errors for the WMW are sensitive to differing shapes and variances between populations [2].

Our reply: We agree that the WMW test is sensitive to differing shapes and variances. However, this is a powerful argument against omission of tied values. In the previous as well as the revised versions of our manuscript we write that “ties are more likely where the density of the distribution in higher. To omit tied values can thus produce a considerable change in the shape of distribution of the remaining data, compared to the whole data set.”

To clarify this further, in the revised version we now add (see page 4) that “as ties are non-randomly distributed in practice, the possible change in the shape of distribution of the remaining data after the omission of ties might lead to changes between groups in skewness, variance and other characteristics that can violate the shift alternative framework”.

Thus, from these arguments, the sensitivity to differing shapes and variances can be regarded as a further argument against the omission of ties.

To address point 1, it has been suggested that the WMW test itself is not practical or realistic [2] (and references within). The WMW, as originally envisioned by Wilcoxon [3], addressed the null and alternative hypotheses 𝐻_0_: 𝜇_1_ = 𝜇_2_ 𝑣𝑠. 𝐻_A_: 𝜇_1_ = 𝜇_2_ + 𝛿, where 𝛿 ≠ 0 is a shift in the location of one of the distributions. The shift alternative implies that the two distributions must be equal in all respects except for the shift in location. In fact, it has been shown through extensive simulations, that “small differences in variances and moderate degrees of skewness can produce large deviations from the nominal Type I error rate” [2]. I suspect that this is part of the reason that Neuhäuser and Ruxton’s results contradict [1]. Their simulations likely produce situations where the shapes and skewness of the two populations being tested violate the shift alternative framework.

Our reply: As mentioned directly above, the omission of ties can change the distributions. This change occurs because ties are more likely where the density of the distribution being sampled is higher. This is the case both when ties occur due to imprecise measurements (rounding) and in the case of discrete distributions. In contrast, in McGee’s simulation randomly chosen values are regarded as ties and omitted. Under their methodology, omission of ties does not alter the distribution of the data. Thus, this can indeed be a reason for the different results. However, we consider that McGee’s approach to declare randomly chosen values as “ties” does not correspond to real-world situations. We do not make this criticism directly in our manuscript, because it is not essential to our arguments to criticise McGee’s simulations in this way. We do however now draw the reader’s attention to this difference between our approaches.

Secondly, as Neuhäuser and Ruxton point out, the position of the ties within a sample can make a difference in Type I error for the WMW [4]; therefore, random placement of ties is unrealistic. The result in [4] gives bounds on Pitman efficiencies given the placement of compliers and noncompliers within samples in an intention to treat analysis for both the WMW and the TST. I am glad to know about this result. However, [1] considered that the position of ties would affect the Type I and Type II errors by randomly distributing ties in terms of their position within a sample. In the context of experimental design, randomization to mitigate the effects of unexamined factors theoretically and practically justified [5]. Randomization is an experimental tool that mitigates the effect of confounding variables that are unknown or unnamed within an experiment.

As quoted in Fisher, 1926 [6]:

One way of making sure that a valid estimate of error will be obtained is to arrange the plots deliberately at random, so that no distinction can creep in between pairs of plots treated alike and pairs treated differently; in such a case an estimate of error, derived in the usual way from the variation of sets of plots treated alike, may be applied to test the significance of the observed difference between the averages of plots treated differently.

Our reply: Indeed, we agree that random placement of ties is unrealistic (as we discuss directly above). The frequency of ties is not a factor a researcher can set as part of experimental design. However, the average frequency of ties is influenced by the precision of measurements, which is reflected in our simulation where we round to differing number of decimal places.

Thirdly, [1] advocates omitting ties only when the percentage of ties in the sample is less than 15%. Figure 5 [1] shows that the WMW has Type I error close to nominal only if the sample sizes are equal, the percentage of ties is 10% or 15%, and only when the the sample sizes for both groups are greater than 10. Therefore, omitting ties should be done when all these stipulations are met.

Our reply: We could not find a statement that McGee (2018) advocates omitting ties only when the percentage of ties in the sample is less than 15%. The abstract says “.... therefore, we recommend omitting tied observations when they occur for both the two-sample t-test and the Wilcoxon-Mann-Whitney.” A similar statement is given in McGee’s conclusions (page 16). Result number 2 on page 15 states “Omitting tied observations has the best Type I error and power properties of the four methods of tie adjustment considered, and this effect is true for the TST as well as the WMW.”

However, we added the following text on page 6 of our revised manuscript: “McGee (2018) reported a more erratic, or worse, type I error rate and a reduced power when tied observations occur with a percentage 25% or 50% and are omitted. On the basis of those results, one might advocate omitting ties only when the percentage of ties in the sample is less than 15%. However, such a strategy would often preclude defining the statistical approach ahead of data collection, since the percentage of ties would normally be difficult to predict with any confidence. Further, the exact test with tied observations included has preferable type I error and power values than the test performed after omission of ties in the scenarios presented in Tables 1 and 3 where the average proportion of ties is not larger than 10%. Thus, the exact permutation test is always a good choice.”

Lastly, there is a fundamental difference in the way [1] and Neuhäuser and Ruxton perform simulations that indicate inflated Type I error when ties are omitted from the samples. Neuhäuser’s and Ruxton’s simulations are done in a context where random variables are generated from one of several distributions and then the continuous values are rounded to one decimal place. In their simulations, the WMW test is performed only on the unique values in the data set, which effectively eliminates all tied values. The simulation set up forces the percentage of ties within and between samples to be subject to the observations within each random sample. In essence, the percentage of ties cannot be predicted from the outset, which is the definition of random.

Our reply: Yes, the percentage of ties is not constant in our simulations which, in our opinion, exactly reflects the situation in applications when ties occur due to imprecise measurements (rounding) as well as in the case of discrete distributions.

I used Neuhäuser’s and Ruxton’s available code to perform simulations only for the Normal case where the sample sizes are equal at n = m = 10 and 𝜇_1_ = 𝜇_2_ = 0. I used 10000 iterations and the same seed that was provided in the code. My Type I error in the continuous case (all values included) was 0.043 and in the discretized case (ties were present) it was 0.0471, both of which are close to the nominal significance level of 0.05. However, I modified the code somewhat to store the p-values (for both cases), the number of observations kept, and the number of observations in each sample for all 10000 iterations. My modified code is given at the end of this review. A histogram (left) and an empirical distribution function (right) of the percentage of observations deleted, which is equivalent to the percentage of tied observations, is given in Figure 1.

 Figure 1: The left panel shows a histogram of the percentage of omitted observations for the case when values from a continuous random normal distribution with mean 0 and variance 1 are rounded to one decimal place, thus forces tied observations. The starting sample size is n = m = 10. The right panel shows an empirical distribution function of the same data.

The center of the histogram is approximately 0.4, which indicates that the average percentage of observations omitted in this simulation is 40%. However, the percentage could be as low a 0% or as high as 90%. The five-number summary for the percentage of tied observations is 0, 30, 40, 50, 90. In other words, at least 25% of the simulations from Neuhäuser and Ruxton have 30% of the observations tied, and at least 75% of them have 50% of the observations tied. This is shown graphically in Figure 1(b) with the empirical distribution function. For the ECDF, the percentage of omitted observations is plotted on the horizontal axis and cumulative proportion for each observation is given on the vertical axis. The simulations have a much greater percentage of tied observations than the simulations in [1], where the percentage of tied observations was kept constant for various scenarios.

Neuhäuser and Ruxton need to address this issue in a revision.

Our reply: As mentioned above, it is our strong contention that the frequency of ties is not a factor researchers can fix in their experimental design. This is reflected in our simulations: we have one scenario with few ties (rounding to two decimal places, Tables 1 and 3) and one scenario with more frequent ties (rounding to one decimal places, Tables 2 and 4). We can confirm the average percentage of ties of approx. 40% in the simulation reproduced by McGee, see first results line 1 in Table 2: the average sample size is 11.9 which corresponds to 11.9/20 ≈ 60% non-tied observations.

Our means of generating tied values in our simulated samples is different from that used by McGee. As we have discussed above, we firmly believe that our method is a much more realistic representation of the processes that generate ties in real data. In the revised manuscript we added the text (see page 5): “Thus, in McGee’s simulations the percentage of tied observations was kept constant in the different scenarios. In our simulations, the number of ties varies, which we believe better reflects the situation in statistical practice.”

Finally, in focusing on the WMW test and the assignment of ties, Neuhäuser and Ruxton miss some of the main ideas of [1]. [1] examined multiple replacements for tied observations, including omission, jittering, mid-ranks, and average scores. Simulations for 15 different pairs of equal sample sizes, ranging from n = m = 5 to n = m = 20, and 14 different sets of unequal sample sizes (starting with n = 6 and m = 3 and ranging to n = 20 and m = 18) were performed. Four types of adjustments for ties were compared using 4 distributions and 4 different percentages of tied observations for both the WMW and the TST. This is a total of 2[(15 × 4 × 4 × 4) + (14 × 4 × 4 × 4)] = 3720 simulations. These simulations were done for the situation where 𝜇_1_ = 𝜇_2_ = 0 (Type I error). Additional simulations were done to determine Type II error. Neuhäuser and Ruxton’s results address Type I error and Type II error only for omitting ties, and not for other common methods of correcting for tied observations.

Our reply: Some other methods were investigated by McGee (2018) but were not recommended by her. Therefore, we did not include them in our simulations. However, we added a paragraph (see page 7 of the revised manuscript): “It should be noted that McGee (2018) also investigated other methods of handling ties. Her detailed study shows some advantages and disadvantages of the various procedures. McGee (2018) concluded that, aside from omission of tied observations, the next best option would be jittering the data, i.e. adding random noise to the observations to break ties. However, an exact permutation test with mid-ranks is preferable to using randomly broken ties (Tilquin et al., 2003; Neuhäuser and Ruxton, 2009; Neuhäuser, 2012).”

In reviewing this paper, I thought of an additional reason that the placement of ties could affect Type I and Type II error. Perhaps the difference can be thought of as ties “within” samples vs. ties “between samples”. To illustrate the difference, consider the following data set from [1].

Suppose we have the following data that represent a score on a scale of alertness of 11 students, five who had consumed 8 ounces of coffee prior to the test, and six who drank 8 ounces of water. For the coffee group, we have scores 21 31 29 27 35, and for the water group, we have 21 19 17 21 20 19.

To begin the ranking process, we put all the scores into one vector: 21 31 29 27 35 21 19 17 21 20 19. The first 5 numbers (in bold red) are from the coffee drinking treatment and the second 5 are from the water drinking treatment. The score of 21 in the coffee treatment and the score of 21 in the water treatment are examples of “between samples” ties, which means that an observation in one sample is tied with an observation in the other sample. The score of 19, appearing twice in the water treatment group, is an example of a within sample tie, because a score of 19 does not appear in the coffee treatment group. Incidentally, the score of 21 is both a between sample tied score and a within sample tied score. [1] focused on between-sample tied scores, as the code randomly selected certain rows with a data frame to be tied, while Neuhäuser and Ruston’s simulations contain both within sample and between sample ties.

Our reply: That is indeed an important point. McGee (2018) focused on between-sample ties. However, when ties occur in practice, there is no reason that only between-sample ties occur. Within-sample ties are also possible (and even more likely when there are large differences between groups, as in power simulations). Thus, our simulations where both within- and between-sample ties can occur is a realistic scenario.

Within-sample ties can have a large influence when ties are omitted as proposed by McGee (2018). When within-sample ties occur and are omitted, the ratio *n*_1_/*n*_2_ can change. “Unequal sample sizes have a detrimental effect on Type I error and power …, with the effect worsening as the ratio of the sample sizes is larger” (McGee, 2018, point 4 on page 15). In McGee’s simulation an identical number of observations in the two groups were regarded as ties. Thus, when the original sample sizes are balanced with *n*_1_ = *n*_2_, the sample sizes are also balanced after the omission of ties. This would often not be the case in practice, because within-sample ties can occur differently. Hence, after the omission of ties the sample sizes are unbalanced with *n*_1_ ≠ *n*_2_, and this has a detrimental effect. As a consequence, the strategy to omit ties would perform less well in realistic settings than McGee’s simulations indicate.

However, within-sample ties can also have an effect without changing sample sizes as illustrated by the following example (see Neuhäuser and Ruxton, 2009; Neuhäuser, 2012, page 49): It is a fictitious example with *n*_1_ = *n*_2_ = 6. In group 1, the values 1 and 3 are observed three times each. In group 2, we have two times the value 2, three times the value 4, and once the value 5. Thus, there are ties within the groups only. The mid-ranks in group 1 are 2, 2, 2, 7, 7, and 7; hence, the rank sum is 27. The resulting p-value of the exact permutation Wilcoxon-Mann-Whitney test is 0.0498.

When we apply jittering, i.e. we add some random noise to break the ties, the rank sum is unchanged, but the significance (at α = 0.05) is lost, the p-value is now 0.0649. If an asymptotic WMW test is performed, the significance is lost as well. Omitting ties would be of course no useful option in this example data set.

Thus, within-group ties have a large influence. Therefore, a study with a focus on between-group ties cannot lead to reliable recommendations. In our simulation, both within- and between-group ties occur, as in real-world applications.

Of the two results from [1] that Neuhäuser and Ruxton contest, I agree that permutation tests can be used in situations when assumptions for parametric or nonparametric tests are not met. However, permutation tests require either coding ability or the software to perform them, and it is not clear whether many research scientists without statistical training would have access to them. Many research scientists use the tests that are available within Excel or menu-driven statistical software. Neuhäuser’s and Ruxton’s simulations also have a large percentage of tied observations that are deleted in their simulations. [1] recommended that omitting ties is valid only if the percentage of ties is less than 15% and the sample size is reasonable. The percentage of tied observations was held constant across simulations while varying other factors to examine the effects of each factor separately. Further, random placement of ties has justification in the experimental design literature [6] for mitigating the effect of the placement of ties within the sample.

Our reply: The exact Wilcoxon-Mann-Whitney test can easily be carried out with several software packages, and a free and easy-to-use online calculator is also available. In the revised manuscript we write (see page 6): “This test is available in various statistical software packages including the open-source statistical software R. In addition, a free and easy-to-use online calculator that provides the exact permutation Wilcoxon-Mann-Whitney test is available at <https://ccb-compute2.cs.uni-saarland.de/wtest/> (Marx et al., 2016).”

As mentioned above, in our simulations we have one scenario with few ties (rounding to two decimal places, Tables 1 and 3) and one scenario with more frequent ties (rounding to one decimal places, Tables 2 and 4). In the former scenario there are on average less than 10% ties. These ties are created by rounding which reflects a realistic occurrence of ties, see also the discussion above. As mentioned at the end of the revised manuscript (see page 7), “we see no reason to recommend the omission of tied observations when applying the Wilcoxon-Mann-Whitney test, and good reason not to recommend such omission, even when the number of ties is low. Exact testing of the whole dataset regardless of ties is a better option.”

Minor:

It is easier to compare Type I and Type II errors across multiple samples when the results are plotted in an effective visualization than it is compare numbers across tables. I would like to see Neuhäuser’s and Ruxton’s tables presented graphically. Not only would a visualization make it easier to compare responses within their study, but also with the results of other similar studies.

Our reply: We do not see an advantage to present the results in graphs. In contrast, the differences are sometimes small and are therefore better read off from tables.

Page 5, line 90 states, “… is estimated as the number of significance tests divided by the number of performed tests”, I think the authors meant “significant” tests. To be more precise, the sentence should read, “estimated as the number of significant tests for which the p-value is less than the nominal level of significance divided by the number of performed tests”, or, even better “estimated as the number of tests for which the p-value is less than the nominal level of significance divided by the number of performed tests”.

Our reply: Rephrased as suggested.
